# Risk Factors for Health Care–Associated Bloodstream Infections in NICUs

**DOI:** 10.1001/jamanetworkopen.2025.1821

**Published:** 2025-03-25

**Authors:** Julia Johnson, Sudhir Malwade, Sharad Agarkhedkar, Bharat Randive, Uday C. Rajput, Chhaya Valvi, Aarti Kinikar, Tushar B. Parikh, Umesh Vaidya, Abhay Kadam, Basma Ouddi, Rachel M. Smith, Matthew Westercamp, Vidya Mave, Christina Schumacher, Susan E. Coffin, Matthew L. Robinson, Amita Gupta, Yukari C. Manabe, Aaron M. Milstone

**Affiliations:** 1 Division of Neonatology, Department of Pediatrics, Johns Hopkins University School of Medicine, Baltimore, Maryland; 2 Department of International Health, Johns Hopkins Bloomberg School of Public Health, Baltimore, Maryland; 3 Department of Pediatrics, Dr. D. Y. Patil Medical College, Pune, India; 4 Johns Hopkins India, Pune, India; 5 Department of Pediatrics, Byramjee Jeejeebhoy Government Medical College, Pune, India; 6 Division of Neonatology, Department of Pediatrics, King Edward Memorial Hospital, Pune, India; 7 Centers for Disease Control and Prevention, Atlanta, Georgia; 8 Center for Child and Community Health Research, Department of Pediatrics, Johns Hopkins University School of Medicine, Baltimore, Maryland; 9 Division of Infectious Diseases, Department of Pediatrics, University of Pennsylvania, Philadelphia; 10 Division of Infectious Diseases, Department of Medicine, Johns Hopkins University School of Medicine, Baltimore, Maryland; 11 Division of Pediatric Infectious Diseases, Department of Pediatrics, Johns Hopkins University School of Medicine, Baltimore, Maryland

## Abstract

**Importance:**

Neonates requiring intensive care are at high risk of health care–associated infections. In neonatal intensive care units (NICUs) in low-resource settings, the identification of modifiable risk factors can inform targeted prevention strategies to reduce the global burden of neonatal morbidity and mortality.

**Objective:**

To describe the incidence of and the risk factors associated with health care–associated bloodstream infections (BSIs) in NICUs in Pune, India.

**Design, Setting, and Participants:**

This multicenter prospective cohort study enrolled all neonates admitted to 3 NICUs in Pune, India, from May 1, 2017, to July 31, 2019. Neonates were followed up from admission until discharge, transfer, or death. This secondary data analysis included neonates admitted for 3 days or more and was completed on January 31, 2024.

**Main Outcomes and Measures:**

The primary outcome was health care–associated BSIs, defined as a positive blood culture on or after admission day 3. Summary statistics, incidence of health care–associated BSIs, and hazard rate by characteristics of interest were generated. Among neonates admitted for 7 days or longer, the association between antibiotic exposure and infection risk was assessed.

**Results:**

A total of 6410 neonates were admitted for 3 days or longer. The median gestational age was 34 weeks (IQR, 32-37 weeks), and 3560 (55.5%) were male. The incidence of health care–associated BSIs was 6.09 per 1000 patient-days. Most isolates were gram-negative organisms (n = 273 [66.3%]), of which 85.5% (202 of 236 isolates tested) were resistant to third- or fourth-generation cephalosporins and 44.8% (117 of 261 isolates tested) were resistant to carbapenems. The hazard rate of health care–associated BSIs was higher among neonates with central venous catheters, respiratory support, or urinary catheters within 3 days preceding infection. Of 3229 neonates admitted for 7 days or longer, 190 (5.8%) had health care–associated BSIs on or after hospital day 7, with an incidence of 3.22 per 1000 patient-days. Antibiotic exposure during the first week of admission was associated with a nearly 3-fold increase in the risk of health care–associated BSIs (adjusted hazard ratio, 2.82 [95% CI, 1.26-6.32]).

**Conclusions and Relevance:**

In this cohort study of 6410 neonates admitted to 3 NICUs in Pune, India, the risk of health care–associated BSIs was associated with the presence of indwelling devices and prior antibiotic exposure. Future efforts should focus on mitigating the risks associated with indwelling devices and strengthening infection prevention and control and antimicrobial stewardship programs to prevent health care–associated infections.

## Key Points

**Question:**

What risk factors are associated with neonatal health care–associated bloodstream infections (BSIs) in low-resource settings?

**Findings:**

In this cohort study of 6410 neonates admitted for 3 days or longer, the incidence of health care–associated BSIs was 6.09 per 1000 patient-days, and these BSIs were primarily caused by gram-negative organisms. The presence of indwelling devices, such as central venous catheters, respiratory support, or urinary catheters, and early exposure to antibiotics were associated with an increase in the risk of health care–associated BSIs.

**Meaning:**

These findings suggest that the use of indwelling devices and prior antibiotic exposure are associated with increased risk of health care–associated BSIs.

## Teaser

This cohort study describes the incidence of and the risk factors associated with health care–associated bloodstream infections in neonatal intensive care units (NICUs) in Pune, India.

## Introduction

Health care–associated infections in neonates admitted to the neonatal intensive care unit (NICU) are associated with increased mortality, as well as increased length of stay and neurodevelopmental impairment among survivors.^1,2^ In settings with a high prevalence of antimicrobial resistance, these outcomes may be magnified because of fewer treatment options, delayed initiation of appropriate therapy, and increased mortality associated with drug-resistant health care–associated infections.^3,4,5,6,7^

The burden of neonatal health care–associated infections in low- and middle-income countries (LMICs) is uncertain. However, rates of infections among hospital-born neonates have been reported as 3- to 20-fold higher than in high-income countries.^8^ Active surveillance for neonatal health care–associated infections is rare in low-resource settings, so our understanding of the drivers of central-line–associated bloodstream infections is limited. Given the less frequent use of central venous catheters, the risk factors for health care–associated bloodstream infections (BSIs) in LMIC NICUs are likely different from other settings. Identification of neonates at highest risk for health care–associated infection events is paramount to guide the development of targeted interventions to prevent health care–associated infections and, thus, reduce the burden of neonatal morbidity and mortality in LMICs.

We conducted a prospective cohort study to describe the epidemiology of neonatal health care–associated BSIs in 3 NICUs in Pune, India. We previously reported the epidemiology of BSIs in this cohort, as well as in a subset of neonates with central venous catheters (CVCs).^9,10^ We performed this secondary data analysis to describe the epidemiology of and the risk factors for neonatal health care–associated BSIs and to describe the association of prior antibiotic exposure with subsequent risk of health care–associated BSIs.

## Methods

### Study Design and Population

This study used data collected from a multicenter prospective observational cohort study among all neonates admitted to 3 tertiary care NICUs in Pune, India, from May 1, 2017, to July 31, 2019. Study sites included Byramjee Jeejeebhoy Government Medical College, Dr. D. Y. Patil Medical College, and King Edward Memorial Hospital. Details for study sites and cohort design have been described previously.^9,10^ In brief, all NICUs have an open-ward structure and care for inborn and outborn neonates, including extremely preterm infants, as well as the capacity to provide mechanical ventilation, perform neonatal surgery, administer intravenous fluids and medications, and insert and maintain CVCs. The study was approved by the ethics committees of all sites, the Indian Council of Medical Research, and the institutional review board of Johns Hopkins Medicine with a waiver of informed consent granted by all reviewing bodies, as consent was not practically feasible for this study, which enrolled all neonates admitted during the study period. We used the Strengthening the Reporting of Observational Studies in Epidemiology (STROBE) reporting guideline to report our findings.

This analysis included data from all neonates enrolled in the parent cohort study and admitted for 3 days or longer. Neonates were followed up from the date of admission until hospital discharge, transfer, or death. Data for this analysis included baseline demographic characteristics, pregnancy and birth information, clinical characteristics, and daily clinical and microbiology data, including blood, urine, and cerebrospinal fluid (CSF) cultures. All cultures obtained were processed in each hospital’s clinical microbiology laboratory, accredited by the Indian National Accreditation Board for Testing and Calibration Laboratories. Laboratory methods included use of VITEK (bioMèrieux Inc) for organism identification and antimicrobial susceptibility testing, as well as manual methods by agar plate and biochemical workup.

### Study Definitions

The primary outcome was health care–associated BSI, defined as a single positive blood culture on or after the third day of hospital admission. We excluded blood cultures positive for known contaminants, although a single culture positive for coagulase-negative *Staphylococcus* was considered a health care–associated BSI event because of the known role of coagulase-negative *Staphylococcus* in late-onset sepsis among neonates admitted to the NICU.^11,12^ The number of hospital days at risk was defined as starting on the third day of admission. Repeat cultures positive for the same organism within 7 days of an initial positive culture were defined as a single health care–associated BSI episode. Positive cultures for the same organism within 7 days of a positive culture collected during the first 2 days of hospital admission were not considered to be health care–associated BSIs and were excluded.

Demographic and clinical characteristics were collected from the medical record and daily observations of clinical care. Daily observations were not systematically collected on weekends and holidays, resulting in information gaps for 29.2% of patient-days (n = 23 635 of 81 014 patient-days). The majority (83.7% [19 774 of 23 635]) of information gaps were 1 day; 12.3% [2917 of 23 635] were 2 days. Devices and exposures of interest were considered present on unobserved days only if the device or exposure was documented as present both immediately before and immediately after the information gap; for all other unobserved days, the exposure was considered absent.

For each hospital day, exposure to indwelling devices and respiratory support was defined as *presence* during any of the prior 3 hospital days. Prior exposure to breast milk on each hospital day was defined as *use* on any previous hospital day.

Antibiotic exposure on each hospital day was defined as *systemic antibiotic administration* and was explored using 3 measures: (1) any documented antibiotic exposure during the past 3 hospital days; (2) early antibiotic use, defined as antibiotic administration during the first 2 hospital days; and (3) any prior exposure, defined as documentation on any prior hospital day. Early BSI, defined as positive blood culture during the first 2 days of hospital admission, was explored as a potential risk factor for subsequent health care–associated BSIs.

### Statistical Analysis

Summary statistics for demographic, pregnancy, birth, and clinical characteristics of all neonates were generated to characterize the study population. The incidence of health care–associated BSIs was calculated as the number of cases per 1000 patient-days at risk and was reported by pathogen type. Among neonates with multiple health care–associated BSIs, the time to the first health care–associated BSI episode was used to calculate incidence in regression models. Hazard rates of health care–associated BSIs by characteristics of interest were estimated using complementary log-log regression, including time as a smoothed function and cluster robust standard errors to account for correlation within NICU sites. To explore the association of clinical characteristics with health care–associated BSIs independent of birth-weight category, hazard rates were adjusted by birth-weight category. In all models, the statistical significance of each hazard ratio was determined by a 95% CI that did not cross 1 and a 2-sided *P* < .05. *P* values were derived from *z* scores.

For the subcohort of neonates admitted for 7 days or longer, analyses were repeated to estimate the hazard rate of health care–associated BSIs associated with increasing exposure density, which was included as a time-varying discrete variable. Exposure density for each hospital day was defined as the proportion of the total number of prior hospital days with the exposure present. For early antibiotic administration, exposure density was also defined for each additional day of antibiotic administration. Analyses were conducted using Stata, version 16.1 (StataCorp LLC) and completed on January 31, 2024.

## Results

### Characteristics of Study Cohort

**Figure 1.**
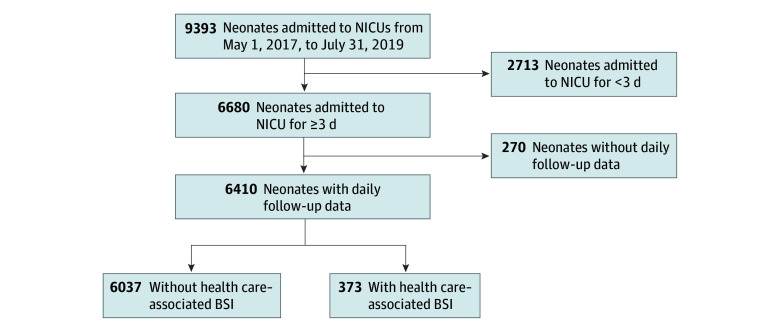
Study Flow Diagram for Analytic Cohort of Neonates Admitted to 3 Tertiary Care Neonatal Intensive Care Units (NICUs) in Pune, India, From May 1, 2017, to July 31, 2019 BSI indicates health care–associated bloodstream infection.

Among 6410 neonates admitted for 3 days or longer with daily observation data, the median gestational age was 34 weeks (IQR, 32-37 weeks), and the median birth weight was 1800 g (IQR, 1400-2440 g); 3560 (55.5%) were male (Figure 1; Table 1). The median length of stay was 7 days (IQR, 4-14 days). Nearly 1 in 10 neonates (9.1%) died before NICU discharge or transfer. Among the 373 neonates (5.8%) with health care–associated BSIs, mortality was 27.1% (n = 101), 60.4% of whom died within 7 days of the health care–associated BSI event.

**Table 1.** Demographic, Birth, and Clinical Characteristics of Neonates Admitted to 3 Tertiary Care Neonatal Intensive Care Units by Health Care–Associated BSI Status in Pune, India, From May 1, 2017, to July 31, 2019

Characteristic	No health care–associated BSI (n = 6037)	Health care–associated BSIs (n = 373)	Total (N = 6410)
Demographic and birth characteristics			
Sex, No. (%)			
Male	3366 (55.8)	194 (52.0)	3560 (55.5)
Female	2668 (44.2)	179 (48.0)	2847 (44.4)
Indeterminate^a^	3 (0.0005)	0	3 (0.0005)
Multiple gestation, No. (%)	796 (13.2)	73 (19.6)	869 (13.6)
Inborn, No. (%)	5375 (89.0)	321 (86.1)	5696 (88.9)
Cesarean delivery, No. (%)^b^	2808 (47.7)	154 (42.8)	2962 (47.4)
Gestational age, median (IQR), wk^c^	34 (32-38)	32 (29-34)	34 (32-37)
Preterm (<37 wk), No. (%)	3682 (66.4)	291 (85.3)	3973 (67.5)
Extremely preterm (<28 wk), No. (%)	185 (3.3)	43 (12.6)	228 (3.9)
Very preterm (28-31 wk), No. (%)	1010 (18.2)	118 (34.6)	1128 (19.2)
Moderate to late preterm (32-36 wk)	2487 (44.9)	130 (38.1)	2617 (44.5)
Full term (≥37 wk), No. (%)	1862 (33.6)	50 (14.7)	191 (32.5)
Birth weight, median (IQR), g^d^^,^^e^	1830 (1450-2500)	1400 (1050-1760)	1800 (1400-2440)
All LBW (<2500 g), No. (%)	4447 (75.0)	326 (88.3)	4773 (76.8)
ELBW (<1000 g), No. (%)	307 (5.2)	57 (15.4)	364 (5.8)
VLBW (1000-1499 g), No. (%)	1274 (21.5)	160 (43.4)	1434 (22.8)
LBW (1500-2499 g), No. (%)	2866 (48.3)	109 (29.5)	2975 (47.2)
Non-LBW (≥2500 g), No. (%)	1483 (25.0)	43 (11.6)	1526 (24.2)
Clinical characteristics			
Any respiratory support, No. (%)	3573 (59.2)	347 (93.0)	3920 (61.2)
Respiratory support, median (IQR), d	3 (2-8)	12 (6-22)	4 (2-9)
Mechanical ventilation, No. (%)	1238 (20.5)	215 (57.6)	1453 (22.7)
Ventilator use, median (IQR), d	3 (2-6)	5 (2-10)	3 (2-7)
Surfactant administration, No. (%)	602 (10.0)	96 (25.7)	698 (10.9)
Nutrition			
Any breast milk, No. (%)	5383 (89.2)	344 (92.2)	5727 (89.3)
Indwelling devices			
CVC, No. (%)	1222 (20.2)	198 (53.1)	1420 (22.2)
CVC use, median (IQR), d	7 (4-9)	9 (6-15)	7 (4-10)
PAL, No. (%)	66 (1.1)	9 (2.4)	75 (1.2)
PAL use, median (IQR), d	2 (1-3)	1 (1-2)	2 (1-3)
PIV, No. (%)	4423 (73.3)	205 (55.0)	4628 (72.2)
PIV use, median (IQR), d	3 (2-7)	5 (2-12)	3 (2-7)
Chest tube, No. (%)	121 (2.0)	16 (4.3)	137 (2.1)
Presence of chest tube, median (IQR), d	3 (2-6)	2 (1-4.5)	3 (1-5)
Urinary catheter, No. (%)	344 (5.7)	69 (18.5)	413 (6.4)
Urinary catheter days, median (IQR)	2 (1-5)	2 (1-7)	2 (1-5)
Nasogastric or orogastric feeding tube, No. (%)	4868 (80.6)	362 (97.1)	5230 (74.4)
Presence of feeding tube, median (IQR), d	5 (2-12)	18 (8-29)	6 (2-13)
Any antibiotic exposure, No. (%)	3715 (61.5)	366 (98.1)	4081 (63.7)
Use of antibiotics, median (IQR), d	5 (3-9)	16 (8-24)	5 (3-10)
Early antibiotic exposure, No. (%)	3203 (53.1)	304 (81.5)	3507 (54.7)
Surgery during admission, No. (%)	174 (2.9)	19 (5.1)	193 (3.0)
Disposition			
Length of stay, median (IQR), d	7 (4-14)	24 (13-36)	7 (4-14)
Died, No. (%)	480 (8.0)	101 (27.1)	581 (9.1)

Abbreviations: BSI, bloodstream infection; CVC, central venous catheter; ELBW, extremely low birth weight; LBW, low birth weight; PAL, peripheral arterial line; PIV, peripheral intravenous line; VLBW, very low birth weight.

^a^ Unknown due to ambiguous genitalia.

^b^ For 6245 neonates.

^c^ For 5885 neonates.

^d^ For 6299 neonates.

^e^ For neonates with missing birth weight, admission weight was used to estimate birth weight if obtained within the first 7 days of life.

### Epidemiology of Health Care–Associated BSIs

**Figure 2.**
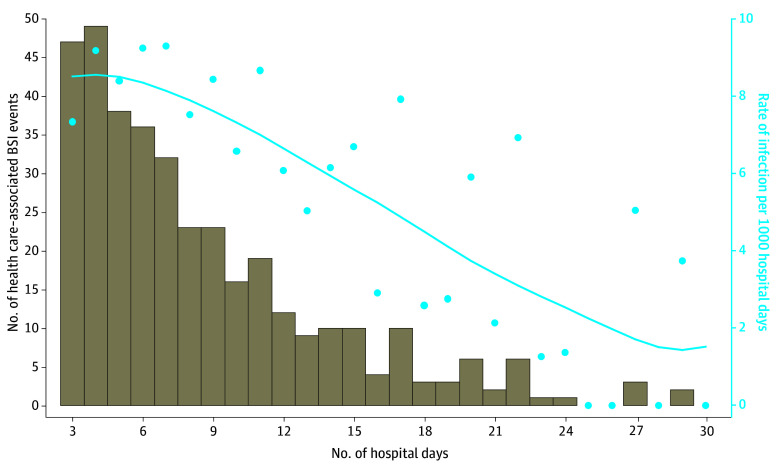
Epidemic Curve of Health Care–Associated Bloodstream Infection (BSI) Events by Hospital Day Among Neonates Admitted for 3 Days or Longer to 3 Tertiary Care Neonatal Intensive Care Units in Pune, India, From May 1, 2017, to July 31, 2019 Bars indicate the number of health care–associated BSI events per hospital day. The plot points indicate the rate of infection per 1000 patient-days for each individual hospital day. The blue curve indicates the locally weighted scatterplot smoothing rate of infection per 1000 patient-days for each hospital day.

There were 409 unique health care–associated BSI episodes among 373 neonates during 61 207 at-risk patient-days (Table 2). The incidence of health care–associated BSIs was 6.09 per 1000 patient-days; the incidence of gram-negative health care–associated BSIs and gram-positive health care–associated BSIs was 4.12 and 1.52 per 1000 patient-days, respectively. The incidence of health care–associated BSIs did not vary significantly by birth-weight category. Most health care–associated BSI events (n = 202 [54.2%]) occurred during days 3 to 7 of admission, and 112 (30.0%) and 38 (10.2%) occurred during the second and third weeks of admission, respectively (Figure 2).

**Table 2.** Hazard Rate of Health Care–Associated BSIs Among Neonates Admitted to 3 Tertiary Care Neonatal Intensive Care Units in Pune, India, From May 1, 2017, to July 31, 2019^a^

Characteristic	No. of health care–associated BSIs	No. of patient-days	Unadjusted HR (95% CI)	Adjusted HR (95% CI)^b^
Demographic and birth characteristics				
Sex				
Female	179	26 582	1.20 (1.04-1.38)^c^	1.15 (1.05-1.26)^c^
Male	194	34 574	1 [Reference]	1 [Reference]
Gestational age				
Extremely preterm (<28 wk)	43	5475	2.45 (1.03-5.83)^d^	1.88 (0.91-3.89)
Very preterm (28-31 wk)	118	21 623	1.30 (0.83-2.06)	1.06 (0.67-1.68)
Moderate to late preterm (32-36 wk)	130	20 367	1.25 (0.94-1.66)	1.17 (0.85-1.60)
Full term (≥37 wk)	50	9588	1 [Reference]	1 [Reference]
Birth weight				
ELBW (<1000 g)	57	9525	1.70 (0.86-3.40)	NA
VLBW (1000-1499 g)	160	23 946	1.34 (0.74-2.14)	NA
LBW (1500-2499 g)	109	19 424	0.97 (0.65-1.46)	NA
Non-LBW (≥2500 g)	43	7405	1 [Reference]	NA
Clinical characteristics				
Respiratory support				
Any respiratory support, past 3 d				
Yes	285	31 088	3.09 (2.86-3.34)^e^	3.16 (2.69-3.72)^e^
No	88	30 119	1 [Reference]	1 [Reference]
Mechanical ventilation, past 3 d				
Yes	89	7944	1.91 (1.58-2.31)^e^	1.90 (1.56-2.33)^e^
No	284	53 263	1 [Reference]	1 [Reference]
Nutrition				
Breast milk, any prior day	288	52 819	0.65 (0.56-0.74)^e^	0.58 (0.54-0.62)^e^
No breast milk	85	8388	1 [Reference]	1 [Reference]
Indwelling devices				
CVC presence, past 3 d				
Yes	143	11 506	2.35 (1.55-3.57)^e^	2.29 (1.24-4.23)^c^
No	230	49 701	1 [Reference]	1 [Reference]
Urinary catheter presence, past 3 d				
Yes	26	1759	2.24 (1.48-3.40)^e^	2.69 (2.02-3.58)^e^
No	347	59 448	1 [Reference]	1 [Reference]
Nasogastric or orogastric feeding tube presence, past 3 d				
Yes	334	51 398	1.61 (0.50-5.15)	1.44 (0.53-3.93)
No	39	9809	1 [Reference]	1 [Reference]
Antibiotic exposure				
Antibiotic exposure, any prior day				
Yes	346	51 642	2.93 (1.50-5.74)^e^	2.70 (1.57-4.65)^e^
No	27	9565	1 [Reference]	1 [Reference]
Antibiotic exposure, past 3 d				
Yes	275	32 080	2.20 (1.61-3.02)^e^	2.20 (1.62-2.99)^e^
No	98	29 127	1 [Reference]	1 [Reference]
Early antibiotic exposure^f^				
Yes	304	45 545	1.80 (0.85-3.80)	1.66 (0.87-3.17)
No	69	15 662	1 [Reference]	1 [Reference]

Abbreviations: BSI, bloodstream infection; CVC, central venous catheter; ELBW, extremely low birth weight; HR, hazard ratio; LBW, low birth weight; NA, not applicable; VLBW, very low birth weight.

^a^ HRs were calculated using complementary log-log regression to estimate the hazard rate of health care–associated BSIs. All models included time as a smoothed function and used cluster-robust standard errors to account for clustering of neonates in neonatal intensive care units.

^b^ Adjusted for birth weight.

^c^ *P* < .01.

^d^ *P* < .05.

^e^ *P* < .001.

^f^ Early antibiotic exposure was defined as antibiotic exposure on hospital days 1 and 2.

### Microbiology of Health Care–Associated BSIs

There were 412 unique bacteria isolated in culture, 273 (66.3%) of which were gram-negative organisms, mostly commonly *Klebsiella* spp (124 [30.1%] of all isolates), *Acinetobacter* spp (n = 47 [11.4%]), and *Citrobacter* spp (n = 41 [10.0%]) (eTable 1 in Supplement 1). Among gram-negative health care–associated BSIs, 85.5% (202 of 236) of tested isolates were resistant to third- or fourth-generation cephalosporins, and 44.8% (117 of 261) were resistant to carbapenems.

### Risk Factors for Health Care–Associated BSIs

An increased hazard rate of health care–associated BSIs was associated with the presence of CVCs (hazard ratio [HR], 2.35 [95% CI, 1.55-3.57]), respiratory support (HR, 3.09 [95% CI, 2.86-3.34]), or urinary catheter (HR, 2.24 [95% CI, 1.48-3.40]) within the 3 preceding days (Table 2). A higher hazard rate of health care–associated BSIs was observed among neonates who received any prior antibiotics (HR, 2.93 [95% CI, 1.50-5.74]) and those who received antibiotics within the prior 3 days (HR, 2.20 [95% CI, 1.61-3.02]). Neonates who received any breast milk had a lower hazard rate of health care–associated BSIs (HR, 0.65 [95% CI, 0.56-0.74]). Observed HRs were similar when adjusting for birth-weight category (Table 2).

### Health Care–Associated BSIs Among Neonates Admitted for 7 Days or Longer

Of the 3581 neonates admitted for 7 days or longer, 282 (7.9%) had a positive blood, CSF, or urine culture before admission day 7 and were excluded from our subanalysis. The remaining 3299 neonates contributed 39 202 patient-days of follow-up time starting on admission day 7. Compared with the full cohort, neonates included in the subcohort were more likely to be preterm, born via cesarean delivery, and exposed to devices and antibiotics but had similar all-cause mortality rates (8.9% [n = 294]) (eTable 2 in Supplement 1).

In the subcohort, 190 neonates (5.8%) had health care–associated BSIs on or after hospital day 7, with an incidence of 3.22 per 1000 patient-days. Mortality was 24.2% (n = 46), and 47.8% (22 of 46) died within 7 days of health care–associated BSI onset. When adjusting for birth weight, health care–associated BSIs were associated with the presence of CVC (adjusted HR, 3.04 [95% CI, 2.50-3.69]), respiratory support (adjusted HR, 1.96 [95% CI, 1.40-2.76]), and antibiotic use in the preceding 3 days (adjusted HR, 2.57 [95% CI, 2.02-3.26]). Any prior exposure to breast milk was associated with a decreased hazard rate of health care–associated BSIs (adjusted HR, 0.52 [95% CI, 0.34-0.80]) (eTable 3 in Supplement 1).

### Association of Antibiotic Exposure With Health Care–Associated BSIs Among Neonates Admitted for 7 Days or Longer

Notably, most neonates included in the subcohort (81.2% [n = 2678]) started antibiotic treatment within the first 7 days of admission; 77.7% of neonates (n = 2082) received antibiotics for 3 consecutive days or more, 58.7% (n = 1571) for 5 days or more, 42.4% (n = 1135) for 7 days or more, and 10.2% (n = 273) for 14 days (eTable 4 in Supplement 1). Of neonates who started antibiotic treatment within 1 day of a negative blood culture (n = 1628), 54.1% (n = 880), 39.1% (n = 636), and 8.9% (n = 144) received antibiotics for 5 or more consecutive days, 7 or more consecutive days, and 14 consecutive days, respectively. Similarly, of 760 neonates without a blood culture, 64.2% (n = 488) received antibiotics for 5 days or more, 45.7% (n = 347) for 7 days or more, and 11.8% (n = 90) for 14 days or more.

Antibiotic use during the first week of admission was associated with a nearly 3-fold increase in risk of health care–associated BSIs (adjusted hazard ratio, 2.82 [95% CI, 1.26-6.32]) on or after hospital day 7 (adjusted HR, 2.96 [95% CI, 1.22-7.17]) when adjusting for birth-weight category. The risk of health care–associated BSIs was associated with an increase in exposure density to devices and antibiotics (Table 3). Each 10% increase in exposure density for antibiotic use (adjusted HR, 1.18 [95% CI, 1.14-1.23]) and each additional day of antibiotic exposure during the first 7 days of admission (adjusted HR, 1.08 [95% CI, 1.02-1.15]) were associated with an increased hazard rate of health care–associated BSIs.

**Table 3.** Adjusted HRs by Increasing Exposure Density Among Neonates Admitted to 3 Tertiary Care Neonatal Intensive Care Units for 7 Days or Longer in Pune, India, From May 1, 2017, to July 31, 2019^a^

Clinical characteristic	Adjusted HR (95% CI)^b^
Respiratory support	
Any respiratory support	1.17 (1.15-1.20)^c^
Mechanical ventilation	1.08 (1.00-1.08)
Indwelling devices	
CVC	1.12 (1.09-1.14)^c^
Urinary catheter	1.14 (1.01-1.29)^d^
Nasogastric or orogastric tube	1.11 (0.67-1.43)
Antibiotic exposure	
Any antibiotic exposure	1.18 (1.14-1.23)^c^
Antibiotic exposure, days 1-7	1.08 (1.02-1.15)^c^

Abbreviations: CVC, central venous catheter; HR, hazard ratio.

^a^ Exposure density was defined as each 10% increase in the proportion of total hospital days with exposure present; for early administration of antibiotics (days 1-7), exposure density is defined as each additional day of antibiotic administration.

^b^ Adjusted HRs represent an increase in the hazard rate per each 10% increase in exposure density and were calculated using complementary log-log regression adjusting for day of admission as a smoothed function and birth-weight category and using cluster-robust standard errors to account for clustering of neonates in neonatal intensive care units.

^c^ *P* < .001.

^d^ *P* < .05.

## Discussion

In this cohort study of 6410 neonates admitted for 3 days or longer to 3 NICUs in Pune, India, nearly 6% developed health care–associated BSIs. More than one-fourth of neonates with health care–associated BSIs died before NICU discharge, with the majority of deaths occurring within a week of health care–associated BSIs. Increased risk of health care–associated BSIs was associated with indwelling devices, including CVCs and urinary catheters, as well as prior antibiotic exposure; nutritional support with breast milk was associated with a decreased incidence of health care–associated BSIs.

Given the high burden of resistant gram-negative health care–associated BSIs, limited treatment options for such infections, and high associated morbidity and mortality, strategies to reduce infection risk among preterm and sick neonates admitted to the NICU are of paramount importance.^9,13,14^ Adaptation of evidence-based strategies to optimize infection prevention and control (IPC) to low-resource settings can be challenging because of barriers such as strained financial resources, staff shortages, supply chain issues, and limitations of the built environment (eg, crowding or inadequate bed spacing, insufficient number of sinks).^15,16,17^ The World Health Organization endorses a multimodal improvement strategy for optimization of IPC practices in health care facilities, with 5 core domains: (1) system change, (2) training and education, (3) monitoring and feedback, (4) reminders and communication, and (5) culture change.^18^ We previously implemented a multimodal IPC improvement strategy, the Comprehensive Unit–based Safety Program, to improve IPC practices and patient safety culture in 4 NICUs in Pune, India, including the 3 study NICUs. Although our intervention did improve IPC practices, including hand-hygiene compliance and aseptic technique for central-line insertion, we did not demonstrate a reduction in rates of health care–associated BSIs.^19^ This and other studies have shown success in improving core IPC practices while still demonstrating the challenges in curtailing the burden of health care–associated infections in LMICs.^20^ In particular, resistant gram-negative infections may not be as amenable to prevention efforts in LMIC settings due to factors such as high rates of colonization, reservoirs of transmission that are challenging to eradicate, and resource shortages. Control strategies for colonized or infected patients may not be feasible in LMIC neonatal units because of a lack of resources for routine admission screening, insufficient personal protective equipment, and typically an open-bay layout without adequate space for isolating or cohorting affected neonates.^21^

Understanding the local context and the unit- and patient-specific risk factors for health care–associated infections is critical to appropriately direct resources to targets with the greatest potential effect. We identified an association between the risk of health care–associated BSIs and the presence of indwelling devices, including CVCs and urinary catheters. Optimization of IPC practices around device insertion and maintenance—as well as timely removal—will be a cornerstone of any neonatal unit IPC program seeking to reduce the risk of health care–associated infection. IPC bundle interventions focused on prevention of device-associated infections have been successful in LMICs.^22^

Among neonates admitted for 7 days or longer, antibiotic exposure during the first week of NICU stay was associated with a nearly 3-fold increase in the risk of health care–associated BSIs. Greater antibiotic exposure intensity (ie, longer duration of antibiotic administration within the first week of admission) was associated with greater risk of health care–associated BSIs. These findings are particularly notable given the frequent use of a prolonged antibiotic course in the absence of positive blood, urine, or CSF cultures. Prolonged antibiotic exposure in the newborn period, although potentially lifesaving, can have serious short- and long-term consequences, such as drug toxic effects, increased risk of necrotizing enterocolitis, late-onset sepsis, retinopathy of prematurity, and disruption of the neonatal microbiome, which may increase risk of chronic illnesses later in life.^23,24,25,26,27,28^

Even with onsite microbiology laboratories, LMIC hospitals may face challenges in obtaining bacterial cultures consistently when medically indicated because of cost, intermittent supply shortages, laboratory equipment requiring repair, and inconsistently staffed laboratories. Clinicians may, therefore, face decisions to continue antibiotics for prolonged empirical courses while cultures are pending or to simply continue for a few more days to complete a treatment course for presumed infection in a neonate whose symptoms and/or risk factors warranted antibiotic initiation and who is clinically improving on antibiotics. As a result, the implementation of antimicrobial stewardship programs that seek to limit antibiotic overuse is challenging in LMICs, given delayed results and the known high burden of resistant bacterial infections among hospitalized neonates.^29^ Prior studies conducted in Indian NICUs have successfully reduced antibiotic overuse without an associated increase in culture-proven sepsis or mortality.^30,31^ These programs include interventions such as requiring documentation of antibiotic indication, prior authorization, de-escalation of therapy, and review and discontinuation of therapy as warranted.^31^ In addition to stewardship interventions implemented directly in the NICU, laboratory capacity building is a critical component of antimicrobial stewardship programs in LMICs. Improving diagnostic capacity will provide key clinician decision-making support for discontinuation and/or de-escalation of antimicrobial therapy, by earlier identification of pathogens and resistance patterns, as well as confirmation of negative cultures with timely communication of these results to the clinical team.

Novel interventions that could potentially reduce infection risk include maternal vaccination for *Klebsiella pneumoniae*, the most common pathogen associated with neonatal health care–associated BSIs in our cohort and a significant contributor to the global burden of neonatal sepsis. In a 2023 bayesian modeling analysis, Kumar et al^32^ estimated that a maternal vaccine with 70% efficacy could prevent nearly 400 000 neonatal sepsis cases and more than 80 000 neonatal deaths globally per year. Neonatal immunity to *K pneumoniae* conferred by maternal vaccination during pregnancy could play a significant role in reducing the risk of nosocomial spread of this pathogen in LMIC NICUs, where *K pneumoniae* is responsible for such a significant proportion of sepsis-associated neonatal morbidity and mortality.^33,34,35^

### Strengths and Limitations

Strengths of our study include its prospective nature, inclusion of multiple neonatal units, and availability of daily patient-level data. Limitations include potential confounding by indication because neonates who require indwelling devices are more likely to be sick and have health care–associated BSIs. We adjusted our analysis for birth-weight category, as low birth weight is associated with increased neonatal morbidity. Our findings may not be generalizable to NICUs in other LMIC settings because all of our study sites were located in the same city in India. However, the epidemiology of health care–associated BSIs in our participating NICUs mirrors that reported from other LMICs, with a predominance of gram-negative organisms, particularly *Klebsiella*, and high rates of antimicrobial resistance. Furthermore, understanding local factors that may influence the risk of health care–associated BSIs is invaluable for the design of future interventions. We may not have captured all health care–associated infections in our population because our microbiologic data were limited to blood, urine, and CSF cultures. Given the challenges in the diagnosis of ventilator-associated pneumonia in neonates, we did not include ventilator-associated pneumonia as a study outcome. It is possible that some of the patients may have had other infections leading to antibiotic use before health care–associated BSIs. We did not collect data on omphalitis, spontaneous intestinal perforation, or necrotizing enterocolitis, although the incidence of omphalitis is exceedingly low at study sites, as are necrotizing enterocolitis and spontaneous intestinal perforation.

## Conclusions

In summary, in our cohort study, neonates admitted for 3 days or longer to 3 NICUs in Pune, India, had a high incidence of health care–associated BSIs, with increased risk among neonates with indwelling devices and prior systemic antibiotic exposure. NICUs should address BSI prevention through improvements in IPC practices, with a focus on risk associated with indwelling devices. It will be important for future studies to explore drivers of high antibiotic use to tailor stewardship programs to local context.

## Notes

Supplement 1. **eTable 1.** Pathogen Distribution for Healthcare-Associated Bloodstream Infections Among Neonates Admitted to Three Tertiary Care Neonatal Intensive Care Units in Pune, India, May 1, 2017-July 31, 2019 **eTable 2.** Demographic, Birth, and Clinical Characteristics of Neonates Admitted to Three Tertiary Care Neonatal Intensive Care Units ≥ 7 Days by Healthcare-Associated Bloodstream Infection (HA-BSI) Status, Pune, India, May 1, 2017-July 31, 2019 **eTable 3.** Hazard of Healthcare-Associated Bloodstream Infection (HA-BSI) Among Neonates Admitted to Three Tertiary Care Neonatal Intensive Care Units ≥ 7 Days, Pune, India, May 1, 2017-July 31, 2019 **eTable 4.** Duration of Antibiotic Courses Initiated During the First Seven Days of Admission Among Neonates Admitted to Three Tertiary Care Neonatal Intensive Care Units ≥ 7 Days, Pune, India, May 1, 2017-July 31, 2019

Supplement 2.
Data Sharing Statement
