# Supplementary material for: Risk Factors for Health Care–Associated Bloodstream Infections in NICUs
Source: JAMA Netw Open. 2025 Mar 25;8(3):e251821. doi: 10.1001/jamanetworkopen.2025.1821 (PMC11937935; doi:10.1001/jamanetworkopen.2025.1821)
Supplement: Supplement 1. — eTable 1. Pathogen Distribution for Healthcare-Associated Bloodstream Infections Among Neonates Admitted to Three Tertiary Care Neonatal Intensive Care Units in Pune, India, May 1, 2017-July 31, 2019 eTable 2. Demographic, Birth, and Clinical Characteristics of Neonates Admitted to Three Tertiary Care Neonatal Intensive Care Units ≥ 7 Days by Healthcare-Associated Bloodstream Infection (HA-BSI) Status, Pune, India, May 1, 2017-July 31, 2019 eTable 3. Hazard of Healthcare-Associated Bloodstream Infection (HA-BSI) Among Neonates Admitted to Three Tertiary Care Neonatal Intensive Care Units ≥ 7 Days, Pune, India, May 1, 2017-July 31, 2019 eTable 4. Duration of Antibiotic Courses Initiated During the First Seven Days of Admission Among Neonates Admitted to Three Tertiary Care Neonatal Intensive Care Units ≥ 7 Days, Pune, India, May 1, 2017-July 31, 2019 [file jamanetwopen-e251821-s001.pdf]

Supplemental Online Content

Johnson J, Malwade S, Agarkhedkar S, et al. Risk factors for health care–associated bloodstream infections in NICUs. *JAMA Netw Open*. 2025;8(3):e251821. doi:10.1001/jamanetworkopen.2025.1821

eTable 1. Pathogen Distribution for Healthcare-Associated Bloodstream Infections Among Neonates Admitted to Three Tertiary Care Neonatal Intensive Care Units in Pune, India, May 1, 2017-July 31, 2019

eTable 2. Demographic, Birth, and Clinical Characteristics of Neonates Admitted to Three Tertiary Care Neonatal Intensive Care Units ≥ 7 Days by Healthcare-Associated Bloodstream Infection (HA-BSI) Status, Pune, India, May 1, 2017-July 31, 2019

eTable 3. Hazard of Healthcare-Associated Bloodstream Infection (HA-BSI) Among Neonates Admitted to Three Tertiary Care Neonatal Intensive Care Units ≥ 7 Days, Pune, India, May 1, 2017-July 31, 2019

eTable 4. Duration of Antibiotic Courses Initiated During the First Seven Days of Admission Among Neonates Admitted to Three Tertiary Care Neonatal Intensive Care Units ≥ 7 Days, Pune, India, May 1, 2017-July 31, 2019

This supplemental material has been provided by the authors to give readers additional information about their work.

© 2025 Johnson J et al. *JAMA Network Open*.

eTable 1. Pathogen distribution for healthcare-associated bloodstream infections among neonates admitted to three tertiary care neonatal intensive care units in Pune, India, May 1, 2017-July 31, 2019

	**Total**<br>**n = 412**<br>n (%)
Gram-negative organisms	273 (66.3)
Klebsiella spp.	124 (30.1)
Acinetobacter spp.	47 (11.4)
Citrobacter spp.	41 (10.0)
Escherichia spp.	20 (4.9)
Enterobacter spp.	16 (3.9)
Pseudomonas spp.	8 (1.9)
Elizabethkingia spp.	5 (1.2)
Serratia spp.	2 (0.5)
Burkholderia spp.	1 (0.2)
Gram-negative, unspeciated	9 (2.2)
Gram-positive organisms	105 (25.5)
Bacillus spp.	11 (2.7)
Enterococcus spp.	12 (2.9)
Staphylococcus spp.	77 (18.7)
Streptococcus spp.	5 (1.2)
Yeast	34 (8.3)
Candida spp.	31 (7.5)
Yeast, unspeciated	3 (0.7)

Table notes. 412 organisms were detected from 409 HA-BSI events among 373 neonates. Three neonates had two organisms detected from the same culture: (*Klebsiella* and *Enterococcus* (n=1); *Escherichia* and *Enterobacter* (n=1); *Klebsiella* and *Citrobacter* (n=1). Percentages were calculated by using 412 isolates as the denominator, rather than 409 HA-BSI events. Abbreviations: HA-BSI – healthcare-associated bloodstream infection; *spp.* – species.

© 2025 Johnson J et al. *JAMA Network Open*.

eTable 2. Demographic, birth, and clinical characteristics of neonates admitted to three tertiary care neonatal intensive care units ≥ 7 days by healthcare-associated bloodstream infection (HA-BSI) status, Pune, India, May 1, 2017-July 31, 2019

	No HA-BSI n=3109	HA-BSI n=190	Total n=3299
**Demographic and Birth Characteristics**			
Male sex, n (%)	1763 (56.7)	102 (53.7)	1865 (56.5)
Multiple gestation, n (%)	531 (17.1)	47 (24.7)	578 (17.5)
Gestational age			
Gestational age in weeks, median (IQR)	33 (31-36)	31 (29-33)	33 (31-35)
Preterm (< 37 weeks), n (%)	2291 (79.4)	161 (91.0)	2452 (80.1)
Extremely preterm (< 28 weeks)	122 (4.2)	28 (15.8)	150 (4.9)
Very preterm (28-31weeks)	834 (28.9)	73 (41.2)	907 (29.6)
Moderate to late preterm (32-36 weeks)	1335 (46.3)	60 (33.9)	1395 (45.6)
Term (≥ 37 weeks), n (%)	594 (20.6)	16 (9.0)	610 (19.9)
Birth weight			
Birth weight in grams, median (IQR)	1563 (1250-2020)	1300 (1010-1600)	1550 (1250-2000)
All LBW (< 2500g), n (%)	2592 (84.8)	175 (92.1)	2767 (85.2)
ELBW (< 1000g)	222 (7.3)	33 (17.4)	255 (7.9)
VLBW (1000-1499g)	1066 (34.9)	94 (49.5)	1160 (35.7)
LBW (1500-2499g)	1304 (42.6)	48 (25.3)	1352 (41.6)
Non-LBW (≥ 2500g), n (%)	466 (15.2)	15 (7.9)	481 (14.8)
Inborn, n (%)	2759 (88.7)	171 (90.0)	2930 (88.8)
Cesarean delivery, n (%)	1566 (51.6)	79 (42.2)	1645 (51.0)
**Clinical Characteristics**			
Respiratory support			
Any respiratory support, n (%)	2356 (75.8)	184 (96.8)	2540 (77.0)
Respiratory support days, median (IQR)	6 (2-11)	16.5 (8-26)	6 (3-12)
Mechanical ventilation, n (%)	927 (29.8)	121 (63.7)	1048 (31.8)
Ventilator days, median (IQR)	4 (2-7)	6 (3-11)	4 (2-7)
Surfactant administration, n (%)	479 (15.4)	57 (30.0)	536 (16.2)
Nutrition			
Any breast milk, n (%)	2999 (96.5)	186 (97.9)	3185 (96.5)
Indwelling devices			
CVC, n (%)	955 (30.7)	115 (60.5)	1070 (32.4)
CVC days, median (IQR)	7 (5-10)	11 (7-18)	7 (6-11)
PAL, n (%)	44 (1.4)	7 (3.7)	51 (1.5)
PAL days, median (IQR)	2 (1-4)	1 (1-2)	2 (1-4)
PIV, n (%)	2463 (79.2)	121 (63.7)	2584 (78.3)
PIV days, median (IQR)	6 (3-11)	6 (2-15)	6 (3-11)
Chest tube, n (%)	105 (3.4)	12 (6.3)	117 (3.5)

© 2025 Johnson J et al. *JAMA Network Open*.

	No HA-BSI<br>n=3109	HA-BSI<br>n=190	Total<br>n=3299
Chest tube presence in days, median (IQR)	4 (2-6)	2.5 (1-4.5)	4 (2-6)
Urinary catheter, n (%)	261 (8.4)	33 (17.4)	294 (8.9)
Urinary catheter days, median (IQR)	2 (1-5)	2 (1-6)	2 (1-5)
Naso- or orogastric feeding tube, n (%)	2,959 (95.2)	188 (98.9)	3,147 (95.4)
Feeding tube days, median (IQR)	9 (5-18)	23.5 (14.5-38.5)	10 (6-19)
Antibiotic exposure			
Any antibiotic exposure, n (%)	2,536 (81.6)	189 (99.5)	2,725 (82.6)
Antibiotic days, median (IQR)	7 (4-11)	21 (12-28)	7 (4-12)
Antibiotic exposure, days 1-2, n	2,117 (68.1)	165 (86.8)	2,282 (69.2)
Antibiotic exposure, days 1-7, n (%)	2,495 (80.3)	183 (96.3)	2,678 (81.2)
Surgery			
Surgery during admission, n (%)	151 (4.9)	14 (7.4)	165 (5.0)
Disposition			
Length of stay in days, median (IQR)	13 (9-22)	30 (20-46)	14 (9-23)
Died, n (%)	248 (8.0)	46 (24.2)	294 (8.9)

eTable 1 notes. Abbreviations: CVC – central venous catheter; ELBW – extremely low birth weight; HA-BSI – healthcare-associated bloodstream infection; IQR – interquartile range; LBW – low birth weight; PAL – peripheral arterial line; PIV – peripheral intravenous catheter; VLBW – very low birth weight

© 2025 Johnson J et al. *JAMA Network Open*.

eTable 3. Hazard of healthcare-associated bloodstream infection (HA-BSI) among neonates admitted to three tertiary care neonatal intensive care units ≥ 7 days, Pune, India, May 1, 2017-July 31, 2019

	HA-BSI	Patient-days	Unadjusted Hazard Ratios		Hazard Ratios Adjusted for Birth Weight	
			**HR**	**95% CI**	**aHR**	**95% CI**
**Demographic and Birth Characteristics**						
Sex						
Male	102	22269				
Female	88	16893	1.14	1.00-1.30	1.11	0.86-6.16
Gestational Age						
Extremely preterm (< 28 weeks)	28	4484	1.64	0.60-4.46	2.30	0.86-6.16
Very preterm (28-31 weeks)	73	16796	1.14	0.63-2.05	1.32	0.78-2.23
Moderate to late preterm (32-36 weeks)	60	11406	1.38	1.14-1.67	1.51	1.33-1.71
Term (≥ 37 weeks)	16	4195				
Birth weight						
ELBW (< 1000g)	33	7878	0.87	0.33-2.27		
VLBW (1000-1499g)	94	17985	1.09	0.52-2.29		
LBW (1500-2499g)	48	9802	1.02	0.65-1.59		
Non-LBW (≥ 2500g)	15	3117				
**Clinical Characteristics**						
Respiratory Support						
Any respiratory support, past 3 days						
Yes	140	18232	3.30	2.66-4.12‡	3.00	2.61-3.44‡
No	50	20970				
Mechanical ventilation, past 3 days						
Yes	40	4202	2.00	1.35-2.95†	1.96	1.40-2.76‡
No	150	35000				
Nutrition						
Breast milk, any prior day	175	37656	0.59	0.31-1.13	0.52	0.34-0.80†
No breast milk	15	1546				
Indwelling devices						
CVC presence, past 3 days						
Yes	76	6680	2.74	2.23-3.37‡	3.04	2.50-3.69‡
No	114	32522				
Urinary catheter presence, past 3 days						
Yes	8	949	1.51	0.37-6.06	1.52	0.44-5.23
No	182	38253				
Naso- or orogastric feeding tube, past 3 days						

© 2025 Johnson J et al. *JAMA Network Open*.

	HA-BSI	Patient-days	Unadjusted Hazard Ratios		Hazard Ratios Adjusted for Birth Weight	
			HR	95% CI	aHR	95% CI
Yes	176	33457	2.04	0.53-7.86	2.07	0.68-6.29
No	14	5745				
Antibiotic Exposure						
Antibiotic exposure, any prior day						
Yes	184	36250	3.10	0.76-12.70	3.01	0.79-11.54
No	6	2952				
Antibiotic exposure, past 3 days						
Yes	135	18019	2.48	1.98-3.11‡	2.62	1.95-3.52‡
No	55	21183				
Early antibiotic exposure (admission days 1-2)						
Yes	165	7446	1.86	0.86-4.03	1.81	0.90-3.65
No	25	31756				
Early antibiotic exposure (admission days 1-7)						
Yes	183	35764	3.06	1.17-7.95*	2.96	1.22-7.17*
No	7	3438				

eTable 2 notes. Hazard ratios were calculated using complementary log-log regression adjusting for day of admission as a smoothed function and using cluster robust standard errors to account for clustering of neonates within NICU sites. Abbreviations: aHR – adjusted hazard ratio; CI – confidence interval; CVC – central venous catheter; ELBW – extremely low birth weight; HA-BSI – healthcare-associated bloodstream infection; HR – hazard ratio; LBW – low birth weight; VLBW – very low birth weight.

* $p < 0.05$, † $p < 0.01$, ‡ $p < 0.001$

© 2025 Johnson J et al. *JAMA Network Open*.

eTable 4. Duration of antibiotic courses initiated during the first seven days of admission among neonates admitted to three tertiary care neonatal intensive care units ≥ 7 days, Pune, India, May 1, 2017-July 31, 2019

	**Duration ≥3 days n=2082**	**Duration ≥5 days n=1571**	**Duration ≥7 days n=1135**	**Duration ≥14 days n=273**	**All n=2678**
Any antibiotic initiation during first 7 days, n (%)	2082 (77.7)	1571 (58.7)	1135 (42.4)	273 (10.2)	2678 (100)
Antibiotic initiation within 1 day of negative blood culture, n (%)	1190 (73.1)	880 (54.1)	636 (39.1)	144 (8.9)	1628 (100)
Antibiotic initiation >1 day before or after a negative blood culture, n (%)	252 (86.9)	203 (70.0)	152 (52.4)	39 (13.6)	290 (100)
Antibiotic initiation without a blood culture, n (%)	640 (84.2)	488 (64.2)	347 (45.7)	90 (11.8)	760 (100)

eTable 3 notes. Duration of antibiotic courses among neonates admitted for at least seven days and started on an antibiotic course during the first week of admission. Neonates with a positive blood, urine, or CSF culture during the first week of admission were excluded from the subcohort. Duration of antibiotic course represents consecutive antibiotic days and could extend beyond the first week of admission; first dose was administered during the first week. Abbreviations: CSF – cerebrospinal fluid

© 2025 Johnson J et al. *JAMA Network Open*.
