# Supplementary material for: Risk Factors for Health Care–Associated Bloodstream Infections in NICUs
Source: JAMA Netw Open. 2025 Mar 25;8(3):e251821. doi: 10.1001/jamanetworkopen.2025.1821 (PMC11937935; doi:10.1001/jamanetworkopen.2025.1821)
Supplement: Supplement 2. — Data Sharing Statement [file jamanetwopen-e251821-s002.pdf]

Data Sharing Statement

Johnson. Risk Factors for Health Care–Associated Bloodstream Infections in NICUs. *JAMA Netw Open*. Published March 25, 2025. doi:10.1001/jamanetworkopen.2025.1821

Data
Data available: No
